# Differential replication efficiencies between Japanese encephalitis virus genotype I and III in avian cultured cells and young domestic ducklings

**DOI:** 10.1371/journal.pntd.0007046

**Published:** 2018-12-18

**Authors:** Changguang Xiao, Chenxi Li, Di Di, Julien Cappelle, Lihong Liu, Xin Wang, Linlin Pang, Jinpeng Xu, Ke Liu, Beibei Li, Donghua Shao, Yafeng Qiu, Weijie Ren, Frederik Widén, Véronique Chevalier, Jianchao Wei, Xiaodong Wu, Zhiyong Ma

**Affiliations:** 1 Department of Swine Infectious Diseases, Shanghai Veterinary Research Institute, Chinese Academy of Agricultural Science, Shanghai, PR China; 2 ASTRE, Univ Montpellier, CIRAD, INRA, Montpellier, France; 3 Department of Virology, Immunobiology and Parasitology (VIP), The National Veterinary Institute (SVA), Uppsala, Sweden; 4 National Diagnostic Center for Exotic Animal Diseases, China Animal Health and Epidemiology Center, Qingdao, PR China; 5 CIRAD, UMR ASTRE, Phnom Penh, Cambodia; 6 Epidemiology and Public Health Unit, Institut Pasteur du Cambodge, Phnom Penh, Cambodia; Faculty of Science, Ain Shams University (ASU), EGYPT

## Abstract

Japanese encephalitis virus (JEV) genotype dominance has shifted to genotype I (GI) from genotype III (GIII) in China as demonstrated by molecular epidemiological surveillance. In this study, we performed a serological survey in JEV-non-vaccinated pigs to confirm JEV genotype shift at the sero-epidemiological level. The average ratio of GI/GIII infection was 1.87, suggesting co-circulation of GI and GIII infections with GI infection being more prevalent in pigs in China. To gain an insight into the reasons for this JEV genotype shift, the replication kinetics of seven recently-isolated JEV isolates including three GI strains and four GIII strains were compared in mosquito C6/36 cells, chicken fibroblast cells (DF-1) and porcine iliac artery endothelial cells (PIEC). We observed that GI strains replicated more efficiently than GIII strains in DF-1 and PIEC cells, particularly in DF-1 cells with titers reaching 22.9–225.3 fold higher than GIII strains. This shows an enhanced replication efficiency of GI viruses in avian cells. To examine this enhanced replication efficiency *in vivo*, young domestic ducklings were used as the animal model and inoculated with GI and GIII strains at day 2 post-hatching. We observed that GI-inoculated ducklings developed higher viremia titers and displayed a comparatively longer viremic duration than GIII-inoculated ducklings. These results conform to the hypothesis of an enhanced replication efficiency for GI viruses in birds. There are 36 amino acid differences between GI and GIII viruses, some of which may be responsible for the enhanced replication efficiency of GI viruses in birds. Based on these findings, we speculated that the enhanced replication of GI viruses in birds would have resulted in higher exposure and therefore infection in mosquitoes, which could result in an increased transmission efficiency of GI viruses in the birds-mosquitoes-birds enzootic transmission cycle, thereby contributing to JEV genotype shift.

## Author summary

Japanese encephalitis virus (JEV) causes encephalitis in humans and reproductive disorder in pigs. The enzootic transmission cycle of JEV is maintained in nature by several species of mosquitoes and vertebrates including birds and pigs. In recent years, JEV genotype I (GI) replaced genotype IIII (GIII) as the dominant genotype in Asian countries. Genotype shift has an impact on disease control, and understanding the reasons for this shift will offer valuable insight into avenues for future disease control. Therefore, we compared the replication efficiencies of GI and GIII viruses *in vitro* and *in vivo*. We observed that GI viruses show higher replication titers in avian cells and higher viremia levels in young domestic ducklings than GIII viruses, suggesting an enhanced replication efficiency of GI viruses in birds. Based on these findings, we speculated that the enhanced replication of GI viruses in birds could provide increased mosquito infection, leading to an increase in the birds-mosquitoes-birds transmission cycle, thereby contributing to JEV genotype shift.

## Introduction

Japanese encephalitis virus (JEV) is a zoonotic flavivirus that causes encephalitis in humans and reproductive disorders in pigs in the Asian pacific region [1,2]. The genome of JEV is single-stranded positive-sense RNA consisting of a short 5’ untranslated region, a single open reading frame, and a longer 3’ untranslated region. The single open reading frame encodes a polyprotein that is subsequently cleaved by both cellular and viral proteases into three structural proteins (capsid (C), pre-membrane/membrane (PrM), and envelope (E)) and seven nonstructural proteins (NS) (NS1, NS2A, NS2B, NS3, NS4A, NS4B and NS5) [3].

The JEV enzootic transmission cycle is maintained in nature by several species of mosquito vectors and vertebrate hosts. JEV is transmitted predominantly by *Culex* mosquitoes, but several other genera may participate in certain circumstances [4]. Mosquitoes transmit JEV from a viremic vertebrate to a susceptible vertebrate including humans, birds, pigs and other mammals by bite. After infection by JEV-infected mosquitoes, many domestic and wild bird species demonstrate varying degrees of viremia. Some of which, including young domestic ducklings and chicks, as well as ardeid wading birds develop a level of viremia sufficient to infect mosquitoes and are thus considered the amplifying hosts for JEV transmission [4–6]. Among mammal species susceptible to JEV infection, pigs are the only mammals responsible for JEV transmission, because JEV-infected pigs develop a level of viremia that remains high enough to infect mosquitoes for up to 4 days [4].

JEV is phylogenetically divided into five genotypes (genotype I to V) based on the nucleotide sequence of the E gene [7,8]. Genotype III (GIII) has historically been the main causative agent of Japanese encephalitis (JE) and was the dominant genotype throughout most of Asia from 1935 through the 1990s. Genotype I (GI) was isolated in Cambodia in 1967 and remained undetectable until 1977 when a new isolate was collected in China. Notably, molecular epidemiological surveillance of JEV isolates collected during the last 20 years revealed that GIII has been gradually replaced by GI, showing a genotype shift with GI as the dominant genotype in Asian countries [9–10].

Previous analysis of the differences between the two genotypes at genetic and epidemiological levels suggested that GI displaced GIII probably by achieving a replication cycle that is more efficient but more restricted in its host range [11]. This hypothesis was partially supported by an observation that a GI isolate has significantly higher infectivity titers in mosquito C6/36 cells than two GIII isolates [10]. It is known that pathogenicity and infectivity vary among JEV strains. We therefore evaluated the prevalence of GI and GIII infection in pigs in China and used seven recently-isolated JEV strains including three GI strains and four GIII strains to compare their replication efficiency *in vitro* and *in vivo*, with the aim of gaining insight into the reasons for the JEV genotype shift.

## Materials and methods

### Ethics statement

All animal experiments were approved by the Institutional Animal Care and Use Committee of Shanghai Veterinary Research Institute (IACUC No: Shvri-po-2016060501) and performed in compliance with the Guidelines on the Humane Treatment of Laboratory Animals (Ministry of Science and Technology of the People’s Republic of China, Policy No. 2006 398).

### Viruses and porcine serum sample collection

Seven recently-isolated JEV strains including three GI strains (SD12, SH2 and SH7 strains) and four GIII strains (SH1, SH15, SH19 and N28 strains) were used in this study. The basic information for these JEV strains is shown in Table 1. All JEV strains were isolated from aborted pigs or mosquitoes during 2015 and 2016 and were passaged fewer than seven times in cultured cells, including three passages for plaque purification. The genotypes of the JEV strains were identified using the sequence of the E gene, as described previously [12].

**Table 1:** JEV strains used in this study.

Genotype	Strains	GenBank No.	Source	Date	Location
**GI**	SD12	MH753127	Pig	Aug. 2015	Shanghai
	SH2	MH753133	*Culex tritaeniorhynchus*	Jun. 2016	Shanghai
	SH7	MH753129	*Culex tritaeniorhynchus*	Aug. 2016	Shanghai
**GIII**	SH1	MH753128	Pig	Jul. 2015	Shanghai
	SH15	MH753130	*Anopheles sinensis*	Jul. 2016	Shanghai
	SH19	MH753131	*Anopheles sinensis*	Jun. 2016	Shanghai
	N28	MH753126	Pig	Aug. 2015	Shanghai

A total of 2272 porcine blood samples were collected from JEV-non-vaccinated pigs at pig farms and slaughterhouses located in 12 provinces in China in 2016, including Jilin, Inner Mongolia, Xinjiang, Qinghai, Ningxia, Hebei, Jiangsu, Shanghai, Hubei, Hunan, Guangdong and Guangxi. Porcine serum samples were stored at -80°C immediately after centrifugation. The detailed information of the porcine serum samples are shown in S1 Table.

### Serological survey of GI and GIII infection in pigs

Serum samples collected from pigs were screened by a commercial enzyme-linked immunosorbent assay (ELISA) kit specific to JEV infection (Wuhan Keqian Biology, Wuhan, China). The seropositive samples were further examined by antibody-sandwich ELISA to distinguish GI and GIII infection, as described previously [13]. Briefly, a 96-well ELISA plate was coated with 100 μl per well of porcine serum diluted at 1:100 as well as the positive and negative serum controls at 37°C for 90 min and was blocked with 5% skimmed milk in phosphate-buffered saline (PBS) containing 0.05% Tween 20. JEV SD12 (GI) and N28 (GIII) viruses were heat-inactivated in a water-bath at 56°C for 30 min and added into the serum-coated wells at 10^5^ plaque forming units (PFU) per well. Following incubation at 4°C overnight, the diluted (1:3000) mouse anti-JEV antibodies were dispensed into each well and incubated at 37°C for 60 min. The bound antibodies were detected with horseradish peroxidase-conjugated goat anti-mouse IgG (Santa Cruz Biotechnology, Santa Cruz, CA, USA) and subsequently with 3,3',5,5'-tetramethylbenzidine. The optical density (OD_450_) of each well was measured at 450 nm. GI and GIII infection were differentiated by comparing the OD_450_ values produced by GI and GIII viruses [13].

### Detection of replication kinetics of JEV in cultured cells

*Aedes albopictus* C6/36 cells were maintained in RPMI 1640 medium (Thermo Fisher Scientific, Carlsbad, CA, USA) supplemented with 10% fetal bovine serum (FBS) (Thermo Fisher Scientific) at 28°C. Chicken fibroblast cells (DF-1) and porcine iliac artery endothelial cells (PIEC) were cultured in Dulbecco’s modified Eagle’s medium (DMEM) (Thermo Fisher Scientific) supplemented with 10% FBS at 37°C in an atmosphere containing 5% CO_2_. For JEV infection, C6/36 cells grown on plates were infected with GI or GIII virus at a multiplicity of infection (MOI) of 0.01 and incubated at 28°C for 2h. Following washing with PBS, the cells were cultured in RPMI 1640 medium containing 2% FBS at 28°C for the indicated times. DF-1 and PIEC cells grown on plates were infected with GI or GIII virus at 0.1 MOI and incubated at 37°C for 2 h. Following washing with PBS, the cells were cultured in DMEM supplemented with 2% FBS at 37°C for the indicated times. The supernatants were sampled at the indicated intervals and stored at -80°C. The JEV titers in the supernatants were measured by 50% tissue culture infectious dose (TCID_50_) assay, as described previously [14].

### Detection of viremia in JEV-inoculated ducklings

Specific-pathogen-free Shaoxing ducklings (*Anas platyrhyncha var*. *domestica*) purchased from Harbin Veterinary Research Institute, Chinese Academy of Agricultural Sciences, were inoculated with JEV strains at day 2 post-hatching. Briefly, the ducklings were divided randomly into GI and GIII strain-inoculated groups (*n* = 10) and inoculated subcutaneously with 10,000 PFU of JEV per animal [5]. After inoculation, all ducklings were monitored for 7 days and blood samples (0.15 ml) were taken from the jugular vein once daily from 2 days post-inoculation (dpi) to 7 dpi for detection of viremia. The levels of viremia were measured by TCID_50_ assay, as described previously [14].

### Multiple sequence alignment

The amino acid sequences of JEV strains were obtained from GenBank (S2 Table). Multiple sequence alignments were performed using the DNASTAR Lasergene 7.1 (MegAlign). Phylogenetic tree was generated by the neighbor-joining method using MEGA version 6.06.

### Statistical analyses

Student’s *t*-test or two-way analysis of variance (ANOVA) were used for significance analysis. A *p* value of <0.05 was considered significant.

## Results

### GI infection is dominant in pigs

**Fig 1:**
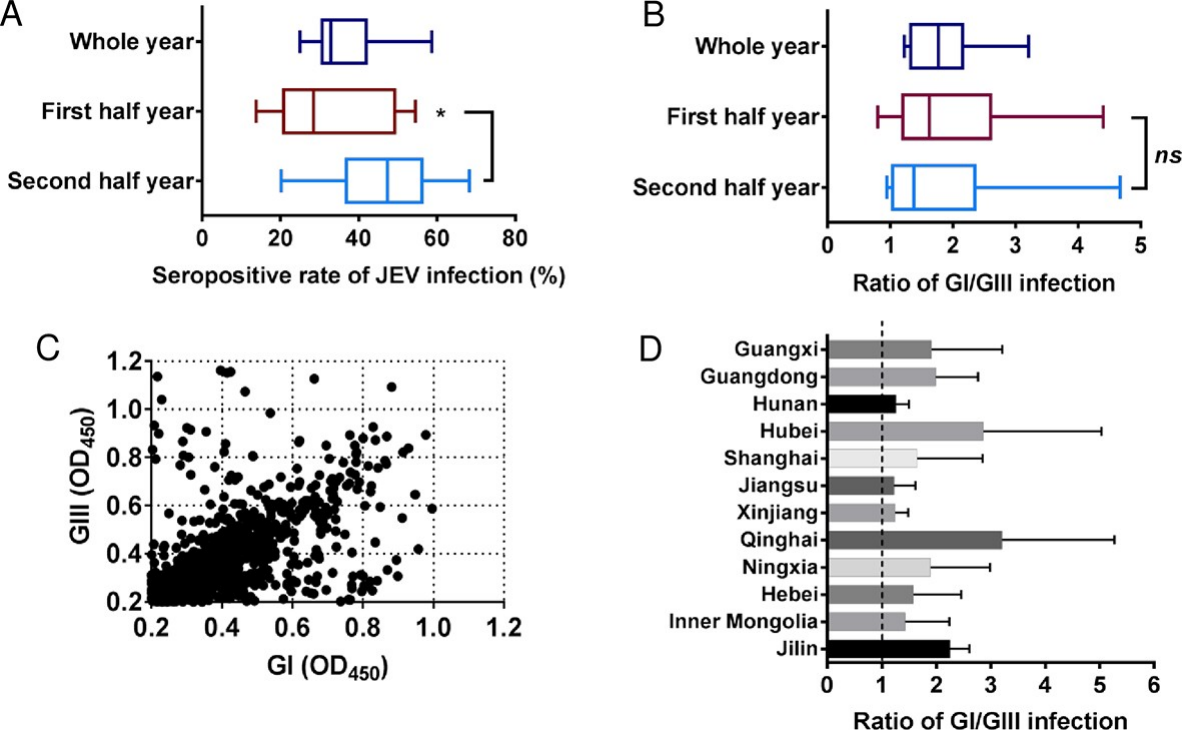
Serological survey of GI and GIII infection in pigs. (A) Seropositive rate (%) of porcine serum samples. *, *p*<0.05. (B) Ratio of GI/GIII infection. *ns*, no significant difference. (C) Distribution of OD_450_ values generated from GI and GIII viruses by the antibody-sandwich ELISA. (D) Ratio of GI/GIII infection at province level.

Although JEV genotype shift has been demonstrated by molecular epidemiological analysis of JEV isolates in China [9,15], the serological evidence for the genotype shift in both humans and pigs was lacking in China. Pigs excluding those used for breeding are not vaccinated for JEV in China and thus are an ideal model for the serological survey and prevalence detection of GI and GIII infection. A total of 2272 serum samples were collected from JEV-non-vaccinated pigs at pig farms or slaughterhouses located in 12 provinces, of which 854 samples were seropositive for JEV infection, as screened by the ELISA kit (S1 Table). The prevalence of JEV infection varied among the 12 provinces, ranging from 25.0% to 58.7% with an average prevalence of 37.6% (Fig 1A and S1 Table). This prevalence rate was in line with a previous surveillance study [15]. The average prevalence of JEV infection in the second half year was 47.0% significantly higher than (32.0%) in the first half year (*p* = 0.0179) (Fig 1A), suggesting an increased prevalence of JEV infection after mosquito season.

To distinguish between GI and GIII infection in the seropositive samples, a previously established antibody-sandwich ELISA was performed, which categorized GI and GIII infection by comparing the OD_450_ values between GI and GIII viruses [13]. A sample producing a greater OD_450_ value against GI virus than GIII virus was considered as GI infection, reversely, a sample producing a greater OD_450_ value against GIII virus than GI virus was classified as GIII infection. Among the 854 seropositive samples, 116 were undistinguishable because their OD_450_ values were nearly identical. Of the remaining 738 samples, 443 and 295 were classified into GI and GIII infection, respectively, with an average GI/GIII infection ratio of 1.87 (Fig 1B and 1C and S1 Table), showing co-circulation of GI and GIII infections with GI infection being dominant in pigs.

No significant difference in the ratio of GI/GIII infection was detected between the first and second half years (Fig 1B). The prevalence of GI and GIII infections was further analyzed at the province level. The ratio of GI/GIII infection varied among the 12 provinces, with the highest ratio of 3.21 in Qinghai and the lowest ratio of 1.23 in Jiangsu (Fig 1D). Taken together, these data indicated that GI infection was dominant for pigs in China, confirming the genotype shift suggested by the molecular epidemiological analysis of JEV isolates.

### GI viruses replicates at significantly higher levels than GIII viruses in chicken and porcine cells

**Fig 2:**
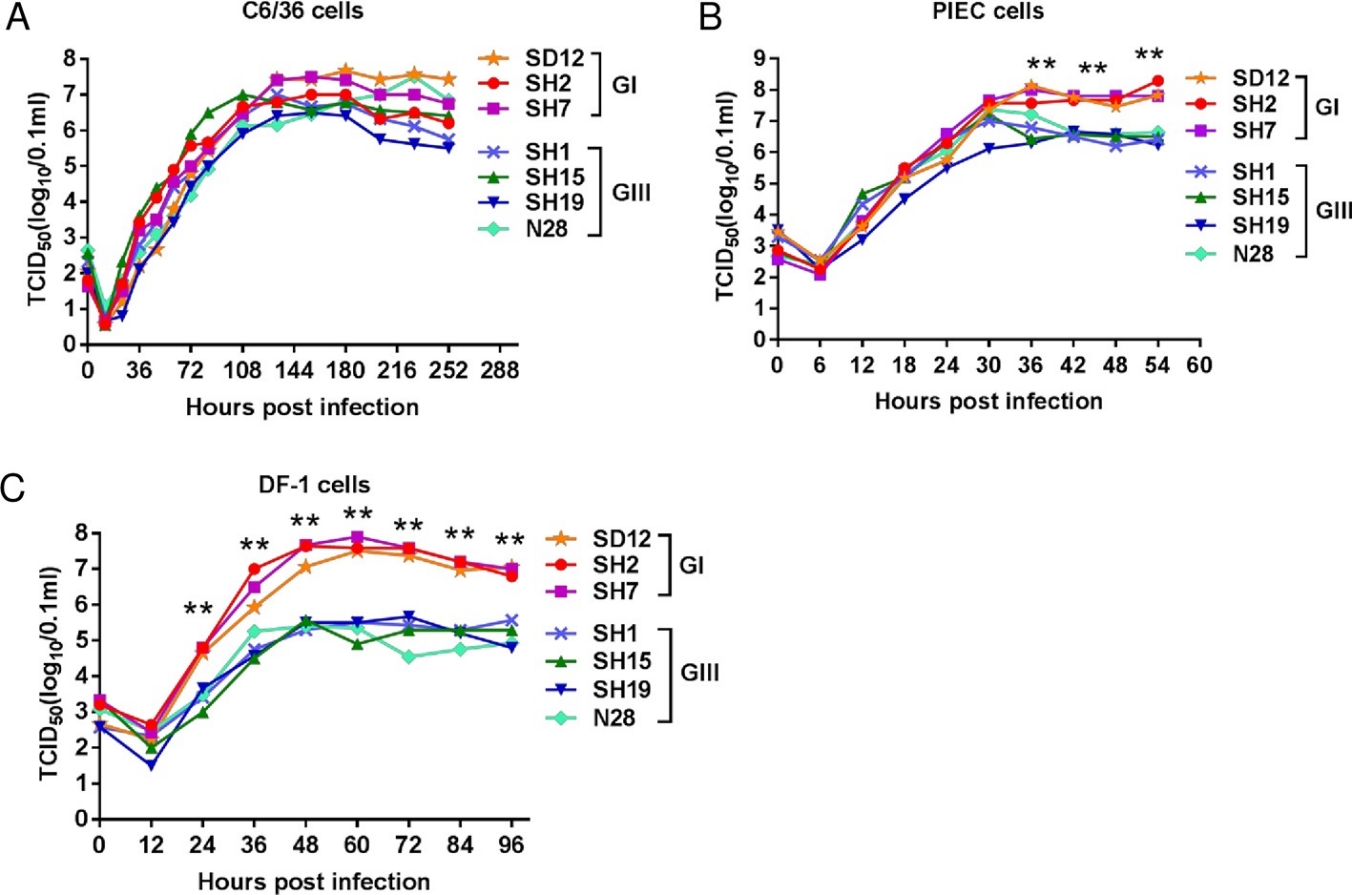
Replication kinetics of GI and GIII strains in cultured cells. (A) Mosquito C6/36 cells. (B) Porcine iliac artery endothelial cells (PIEC). (C) Chicken fibroblast cells (DF-1). The supernatants were sampled at the indicated time points and the replication titers in the supernatants were measured by TCID_50_ assay. **, *p*<0.001 tested by two-way analysis of variance (ANOVA).

Mosquitoes, birds and pigs are the primary hosts of JEV and play essential roles in maintaining the JEV transmission cycle [4]. Previous studies hypothesized that GI displaced GIII by achieving an increased replication efficiency in various hosts [10,11]. Thus, we compared the replication kinetics of GI and GIII viruses in mosquito C6/36 cells, chicken DF-1 and porcine PIEC cells. These cells were susceptible to JEV infection and used in this study as *in vitro* cell models of mosquito, bird and pig hosts, respectively. The C6/36, DF-1 and PIEC cells were inoculated with seven recently-isolated JEV strains including three GI strains (SD12, SH2 and SH7 strains) and four GIII strains (SH1, SH15, SH19 and N28 strains) and their replication titers in the supernatants were measured. No significant difference in replication titers for GI versus GIII were observed in C6/36 cells (Fig 2A). However, notable differences were observed in PIEC and DF-1 cells between GI and GIII strains. GI strains showed significantly higher replication titers than GIII strains in PIEC cells at 42 (*p*<0.0001), 48 (*p* = 0.0003) and 54 hours post-infection (hpi) (*p* = 0.0002) (Fig 2B). More significant differences in replication titers between GI and GIII strains were observed in DF-1 cells (Fig 2C). The average replication titers of the GI strains were 22.9, 51.0, 103.3, 225.3, 192.5, 97.7 and 63.4 fold higher than those of the GIII strains at 24 (*p* = 0.0005), 36 (*p* = 0.003), 48 (*p*<0.0001), 60 (*p*<0.0001), 72 (*p* = 0.0005), 84 (*p*<0.0001) and 96 hpi (*p* = 0.0004), respectively. These data indicate that GI viruses have an enhanced replication efficiency in chicken and porcine cells when compared with GIII viruses.

### GI-inoculated ducklings developed higher viremia levels than GIII-inoculated ducklings

**Fig 3:**
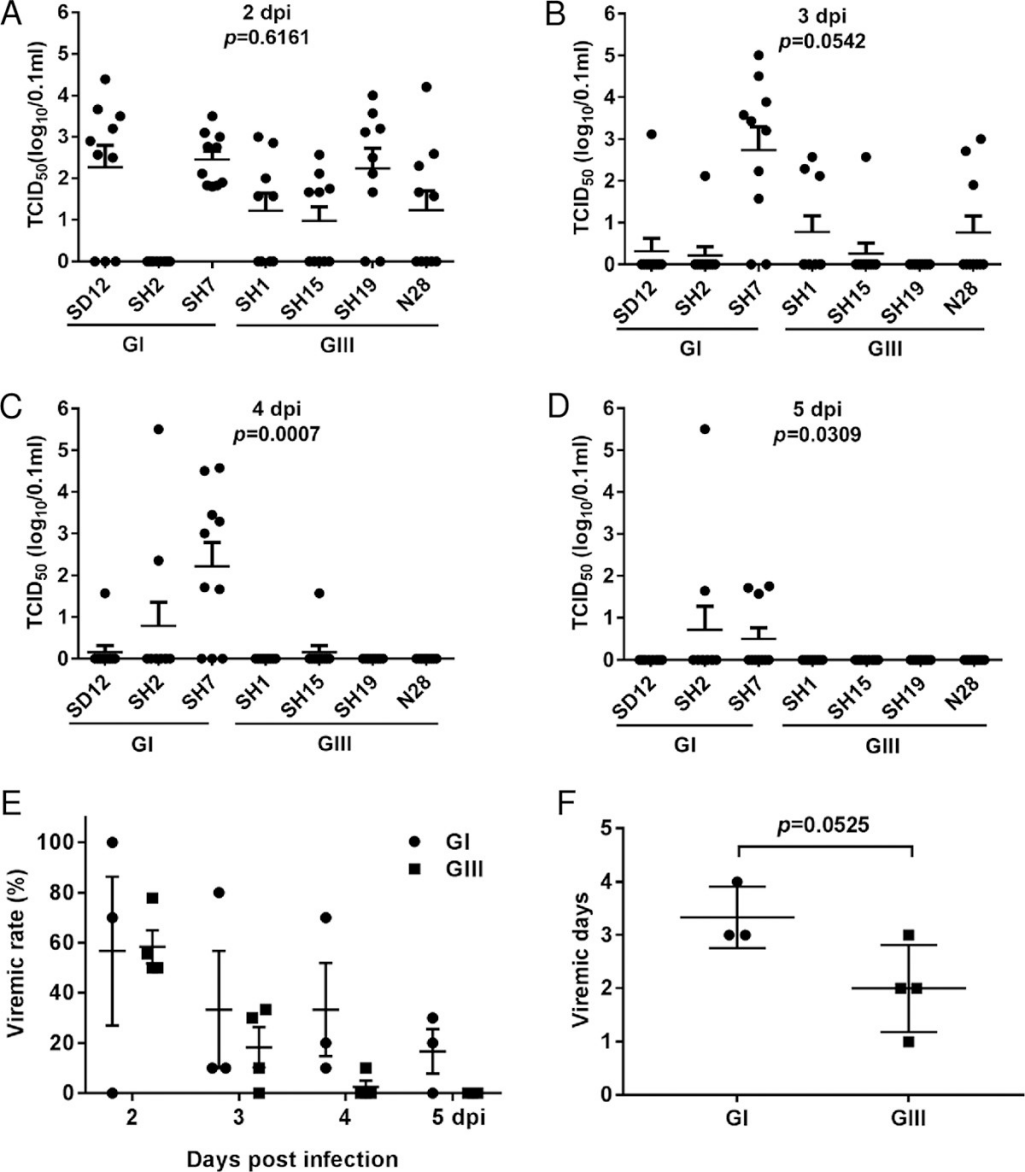
Detection of viremia in JEV-inoculated ducklings. Ducklings (*n* = 10) were inoculated with GI and GIII strains. Blood samples were collected, and the viremia levels measured at 2 dpi (A), 3 dpi (B), 4 dpi (C) and 5 dpi (D) by TCID_50_ assay. No viremia was detectable at 6 and 7 dpi. (E) The viremia rates of GI- and GIII-inoculated ducklings were calculated and plotted. (F) The viremic days were compared between GI- and GIII-inoculated ducklings and plotted. The *p* values were generated by Student’s *t*-test.

Given that the enhanced replication of GI strains was observed in chicken cells, we wanted to determine whether this enhanced replication was repeated in an animal model. Young domestic ducklings develop a detectable level of viremia after JEV infection and are considered an amplifying host contributing to the JEV transmission cycle [5,16]. We therefore used young domestic ducklings as an animal model to compare the replication efficiency between GI and GIII strains. Shaoxing ducklings were subcutaneously inoculated at day 2 post-hatching with JEV strains including three GI strains (SD12, SH2 and SH7 strains) and four GIII strains (SH1, SH15, SH19 and N28 strains), and the levels of viremia were measured. Most of the JEV-inoculated ducklings developed a detectable viremia level starting from 2 or 3 dpi and remained viremic for 1–4 days depending on the strain (Fig 3). No significant difference in viremia levels between GI- and GIII-inoculated ducklings were observed at 2 and 3 dpi (Fig 3A and 3B); however, significant differences were detected at 4 and 5 dpi. GI-inoculate ducklings developed significantly higher viremia levels than GIII-inoculated ducklings at 4 (*p* = 0.0007) and 5 dpi (*p* = 0.0309) (Fig 3C and 3D). The viremic rates were similar between GI and GIII-inoculated ducklings (Fig 3E), while the viremic duration of GI-inoculated ducklings was notably, but not significantly (*p* = 0.0525), longer than GIII-inoculated ducklings (Fig 3F). These data indicate that GI-inoculated ducklings develop higher viremia levels than GIII-inoculated ducklings, suggesting an enhanced replication efficiency of GI viruses in birds.

### Amino acid variations between GI and GIII viruses

Minor mutations in JEV proteins are associated with changes in JEV replication and host fitness [17,18], we therefore performed a multiple alignment on amino acid sequences to detect the amino acid variations between the GI and GIII strains. The amino acid sequences of 19 GI strains and 20 GIII strains (S2 Table) were downloaded from GenBank and the amino acid variations were compared. There are 36 amino acid differences present in viral proteins including three structural proteins (C, PrM and E) and seven nonstructural proteins (NS1, NS2A, NS2B, NS3, NS4A, NS4B and NS5) (Table 2 and S1 Fig), some of which may be responsible for the difference in replication efficiency between GI and GIII viruses.

**Table 2:** Amino acid variations between GI and GIII viruses.

**Viral protein**	**C**				**PrM**		**E**			
**Position number**	100	110	120	122	184	185	423	516	621	660
**GI strains**	K	S	I	T	A	V	M	S	T	S
**GIII strains**	R	G	V	I	T	M	T	A	S	A
**Viral protein**	**NS1**						**NS2A**			
**Position number**	845	864	941	1000	1092	1152	1243	1295	1297	1333
**GI strains**	Q	S	R	L	I	I	A	T	A	R
**GIII strains**	K	A	H	Y	V	V	T	S	T	K
**Viral protein**	**NS2B**		**NS3**					**NS4A**	**NS4B**	
**Position number**	1428	1438	1472	1582	1681	1686	1689	2233	2345	2390
**GI strains**	D	E	L	S	D	S	K	V	S	V
**GIII strains**	E	D/G	V	A	E	N	R	I	N	A
**Viral protein**	**NS5**									
**Position number**	2628	2807	2899	2956	2959	3115				
**GI strains**	K	R	V	G	L	G				
**GIII strains**	R	K	A	D	R	E				

Notably, there were 4, 6, 5 and 6 variations respectively located in the E, NS1, NS3 and NS5 proteins that play important roles in JEV replication. The E protein is the major structural protein containing a receptor-binding domain and neutralization epitopes and plays major roles in mediating virus entry and pathogenicity [17,19]. The amino acid variation in the E protein may influence the cell tropism, penetration into cells and virulence. NS1 is a multifunctional glycoprotein that is present in different cellular locations including the intracellular membranes and cell surface as well as in sera as a secreted lipo-particle, and serves a central role in viral replication, eliciting the immune response, and inhibition of the complement system [20,21]. The amino acid variation in NS1 may also effect its roles in viral replication, immune modulation and immune evasion. NS3 is a multifunctional protein that possesses the enzymatic activities of serine protease, helicase and nucleoside 5’-triphosphatase [22,23]. The amino acid variation in NS3 may result in an altered enzymatic activity in the processing of the viral precursor polyprotein and the replication of viral genomic RNA. NS5 is the largest protein and consists of the methyltransferase (MTase) and RNA-dependent RNA polymerase (RdRP) [24]. MTase is involved in methylation of the 5’ RNA cap structure and RdRP is the key enzyme for viral replication. In addition, NS5 contributes to the blocking of interferon signaling pathways [25]. The amino acid variation in NS5 may change the enzymic activities of MTase and RdRP as well as the antagonization of the interferon response.

## Discussion

Molecular epidemiological surveillance of JEV isolates demonstrated that GI replaced GIII as the dominant genotype in China [9,15,26]; however, this genotype shift has not been confirmed by serological evidence. Vaccination with the SA14-14-2 live attenuated JE vaccine (GIII) results in a difficulty in distinguishing GI and GIII infection in humans using serological surveillance methods. We therefore studied the seroprevalence of GI and GIII infection in JEV-non-vaccinated pigs using the antibody-sandwich ELISA [13] and found that the average ratio of GI/GIII infection was 1.87 among 738 porcine serum samples collected from 12 provinces. These results suggest co-circulation of GI and GIII infections with GI infection dominant in pigs, confirming the genotype shift in China. However, these data generated by the antibody-sandwich ELISA [13] were somewhat provisional and future studies should further validate this approach using sera from animals infected with specific GI and GIII viruses. In addition, there were 116 porcine serum samples undistinguishable for GI and GIII infection, which may be attributable to that the animals were probably infected with both genotype viruses.

Previous analysis of the differences between GI and GIII viruses at genetic and epidemiological levels suggests that GI displaced GIII probably by achieving an increased replication efficiency in hosts [10,11]. To test this speculation, we used seven recently-isolated JEV strains including three GI strains and four GIII strains to compare their replication kinetics in mosquito C6/36, chicken DF-1 and porcine PIEC cells. These viruses were passaged fewer than seven times during isolation and plaque purification to avoid artificial mutations in viral proteins. No significant differences in replication titers were observed in C6/36 cells, but significant differences were observed in DF-1 and PIEC cells, between GI and GIII strains. Particularly, the replication titers of GI strains in DF-1 cells where they were 22.9–225.3 fold higher than those of GIII strains, indicating that GI viruses had higher replication efficiency in avian and porcine cells.

Although both birds and pigs are amplifying hosts in the JEV transmission cycle, JEV genotype shift also occurs in some endemic countries, including India [27], Malaysia [28] and Korea [29], where pig-breeding is not common. This suggests that birds including herons and ducks, but not pigs, are potential hosts contributing to genotype shift [29,30]. It is known that birds, especially wading birds, are considered the primary enzootic hosts of JEV, and play an essential role in epizootic viral amplification in some areas [4]. Young domestic ducklings are susceptive to JEV infection and develop viremia sufficient for mosquito infection [5]. In addition, large numbers of duck farms located near ponds and lakes where mosquitos breed, provide an abundant pool of amplifying hosts for JEV infection and facilitate the birds-mosquito-birds transmission cycle. We therefore used young domestic ducklings as an animal model to confirm the increased replication efficiency of GI strains observed in avian cells. As expected, GI-inoculated ducklings developed higher viremia titers and relatively longer viremia duration than GIII-inoculated ducklings, confirming that the replication efficiency of GI viruses was higher in birds. Taken together, these *in vitro* and *in vivo* data suggest that birds including young domestic ducklings may be the major contributing factor in genotype shift. A previous comparative analysis of replication kinetics between GI and GIII isolates in North American avian species indicated that GI viruses showed higher viremia titers than GIII viruses in several avian species including mallards, house finches and ring-billed gulls [6]. This observation further supports our hypothesis that birds might contribute to JEV genotype shift.

Numerous factors, including the replication efficiency of GI and GIII viruses in hosts, the availability and abundance of amplifying hosts, the contact rates among amplifying hosts and mosquito vectors, and climatic and environmental parameters, are considered to play a role in JEV genotype shift [6,31]. Based on our findings together with the previous observation that GI viruses show higher viremia titers than GIII viruses in several avian species [6], we speculate that the enhanced replication efficiency of GI viruses in birds would have provided mosquitoes more chances to be infected, which led to an increased transmission efficiency of GI viruses in the birds-mosquitoes-birds enzootic transmission cycle, and eventually the displacement of GIII viruses by GI viruses as the dominant genotype.

GI viruses also showed higher replication titers than GIII viruses at the late stage of replication in porcine cells, suggesting that pigs may also be involved in JEV genotype shift. We have inoculated sixty-day old and antibody-negative piglets (*n* = 5) with JEV SD12 strain (GI) and N28 strain (GIII) to examine the difference in viremia development. No significant differences in viremia levels was observed between GI- and GIII-inoculated piglets (S3 Table). However, we could not exclude the role of pigs in JEV genotype shift using this result because of the limited numbers of GI and GIII strains used for experimental inoculation.

A previous observation described a GI isolate (JE-91 strain) showing significantly higher replication titers than GIII isolates (Tiara and Matsunaga strains) in mosquito C6/36 cells in a short period from 24 to 48 hpi [10]. This observation is partially in contrast with our findings where no significant difference in replication titers between GI and GIII strains was observed in mosquito C6/36 cells. However, our findings are in agreement with an *in vivo* observation that no significant differences in replication kinetics and dissemination is observed between mosquitoes infected with the GI isolate (JE-91 strain) and the GIII isolate (Tiara strain) [32]. Our data together with these *in vivo* data suggest that mosquitoes might not play a crucial role in JEV genotype shift.

Multiple alignment of amino acid sequences between GI and GIII viruses indicated that there are 36 amino acid differences present in several viral proteins, including E, NS1, NS3 and NS5, which play important roles in JEV replication, pathogenicity and immune modulation. Minor mutations in JEV proteins are associated with changes in JEV replication and host fitness [17,18]. The amino acid mutations in GI viruses might be responsible for the enhanced replication efficiency of GI viruses in avian cells and young domestic ducklings. This hypothesis is currently under investigation in our laboratory.

In conclusion, a serological survey of JEV infection in JEV-non-vaccinated pigs demonstrated co-circulation of GI and GIII infections with GI infection dominant in pigs in China, despite the fact that these results generated by the antibody-sandwich ELISA were somewhat provisional. Comparative analysis of the replication kinetics of GI and GIII strains indicated that GI strains replicated more efficiently than GIII strains in avian and porcine cells, particularly in avian cells with titers reaching 22.9–225.3 fold higher than GIII strains. In addition, GI-inoculated ducklings developed higher viremia titers and displayed a comparatively longer viremic duration than GIII-inoculated ducklings. These observations suggested that there is enhanced replication efficiency of GI viruses in birds compared with GIII viruses. There are 36 amino acid differences between GI and GIII viruses, some of which may be responsible for the enhanced replication efficiency of GI viruses in birds. Based on these findings, we speculate that the enhanced replication efficiency of GI viruses in birds could provide mosquitoes more chances to be infected, which would lead to an increased transmission efficiency of GI viruses in the birds-mosquitoes-birds enzootic transmission cycle, and eventually the displacement of GIII viruses by GI viruses as the dominant genotype.

## Supporting information

S1 Fig Amino acid variations between GI and GIII viruses. Phylogenetic tree and multiple sequence alignments. The number highlighted in blue indicates the position number of amino acid residue.
(TIF)

S1 Table Information of the porcine serum samples. (XLSX)

S2 Table Information of JEV strains used for multiple sequence alignment. (DOCX)

S3 Table Detection of viremia in JEV-inoculated piglets. (DOCX)

## References

[1] Wang H, Liang G (2015). Epidemiology of Japanese encephalitis: past, present, and future prospects. Ther Clin Risk Manag 11:435–48. 10.2147/TCRM.S51168 eCollection 2015. 25848290 PMC4373597

[2] Ricklin ME, Garcìa-Nicolàs O, Brechbühl D, Python S, Zumkehr B, Posthaus H, et al (2016) Japanese encephalitis virus tropism in experimentally infected pigs. Vet Res 47:34 10.1186/s13567-016-0319-z 26911997 PMC4765024

[3] Unni SK, Růžek D, Chhatbar C, Mishra R, Johri MK, et al (2011) Japanese encephalitis virus: from genome to infectome. Microbes Infect 13:312–321. 10.1016/j.micinf.2011.01.002 21238600

[4] van den Hurk AF, Ritchie SA, Mackenzie JS (2009) Ecology and geographical expansion of Japanese encephalitis virus. Annu Rev Entomol 54:17–35. 10.1146/annurev.ento.54.110807.090510 19067628

[5] Cleton NB, Bosco-Lauth A, Page MJ, Bowen RA (2014) Age-related susceptibility to Japanese encephalitis virus in domestic ducklings and chicks. Am J Trop Med Hyg 90:242–246. 10.4269/ajtmh.13-0161 24394476 PMC3919224

[6] Nemeth N, Bosco-Lauth A, Oesterle P, Kohler D, Bowen R (2012) North American birds as potential amplifying hosts of Japanese encephalitis virus. Am J Trop Med Hyg 87:760–767. 10.4269/ajtmh.2012.12-0141 22927494 PMC3516332

[7] Uchil PD, Satchidanandam V (2001) Phylogenetic analysis of Japanese encephalitis virus: envelope gene based analysis reveals a fifth genotype, geographic clustering, and multiple introductions of the virus into the Indian subcontinent. Am J Trop Med Hyg 65:242–251. 11561712 10.4269/ajtmh.2001.65.242

[8] Solomon T, Ni H, Beasley DW, Ekkelenkamp M, Cardosa MJ, Barrett AD (2003) Origin and evolution of Japanese encephalitis virus in southeast Asia. J Virol 77:3091–3098. 10.1128/JVI.77.5.3091-3098.2003 12584335 PMC149749

[9] Zheng Y, Li M, Wang H, Liang G (2012) Japanese encephalitis and Japanese encephalitis virus in mainland China. Rev Med Virol 22:301–322. 10.1002/rmv.1710 22407526

[10] Schuh AJ, Ward MJ, Leigh Brown AJ, Barrett AD (2014) Dynamics of the emergence and establishment of a newly dominant genotype of Japanese encephalitis virus throughout Asia. J Virol 88:4522–4532. 10.1128/JVI.02686-13 24501419 PMC3993778

[11] Han N, Adams J, Chen P, Guo ZY, Zhong XF, Fang W, et al (2014) Comparison of genotypes I and III in Japanese encephalitis virus reveals distinct differences in their genetic and host diversity. J Virol 88:11469–11479. 10.1128/JVI.02050-14 25056890 PMC4178791

[12] Pan XL, Liu H, Wang HY, Fu SH, Liu HZ, Zhang HL et al (2011) Emergence of genotype I of Japanese encephalitis virus as the dominant genotype in Asia. J Virol 85:9847–9853. 10.1128/JVI.00825-11 21697481 PMC3196406

[13] Fan YC, Chen JM, Chen YY, Lin JW, Chiou SS (2013) Reduced neutralizing antibody titer against genotype I virus in swine immunized with a live-attenuated genotype III Japanese encephalitis virus vaccine. Vet Microbiol 163:248–256. 10.1016/j.vetmic.2013.01.017 23415032

[14] Shi Z, Wei J, Deng X, Li S, Qiu Y, Shao D, et al (2014) Nitazoxanide inhibits the replication of Japanese encephalitis virus in cultured cells and in a mouse model. Virol J 11:10 10.1186/1743-422X-11-10 24456815 PMC3927656

[15] Chai C, Wang Q, Cao S, Zhao Q, Wen Y, Huang X, et al (2018) Serological and molecular epidemiology of Japanese encephalitis virus infections in swine herds in China, 2006–2012. J Vet Sci 19:151–155. 10.4142/jvs.2018.19.1.151 28693301 PMC5799393

[16] Dhanda V, Banerjee K, Deshmukh PK, Ilkal MA (1977) Experimental viraemia and transmission of Japanese encephalitis virus by mosquitoes in domestic ducks. Indian J Med Res 66:881–888. 205503

[17] Tajima S, Nerome R, Nukui Y, Kato F, Takasaki T, Kurane I (2010) A single mutation in the Japanese encephalitis virus E protein (S123R) increases its growth rate in mouse neuroblastoma cells and its pathogenicity in mice. Virology 396:298–304. 10.1016/j.virol.2009.10.035 19913862

[18] de Wispelaere M, Khou C, Frenkiel MP, Desprès P, Pardigon N (2015) A single amino acid substitution in the M protein attenuates Japanese encephalitis virus in mammalian hosts. J Virol 90:2676–2689. 10.1128/JVI.01176-15 26656690 PMC4810689

[19] Liu H, Liu Y, Wang S, Zhang Y, Zu X, Zhou Z, et al (2015). Structure-based mutational analysis of several sites in the E protein: implications for understanding the entry mechanism of Japanese encephalitis virus. J Virol 89:5668–5686. 10.1128/JVI.00293-15 25762738 PMC4442514

[20] Rastogi M, Sharma N, Singh SK (2016) Flavivirus NS1: a multifaceted enigmatic viral protein. Virol J 13:131 10.1186/s12985-016-0590-7 27473856 PMC4966872

[21] Poonsiri T, Wright GSA, Diamond MS, Turtle L, Solomon T, Antonyuk SV (2018) Structural study of the C-terminal domain of nonstructural protein 1 from Japanese encephalitis virus. J Virol 92 pii: e01868-17. 10.1128/JVI.01868-17 29343583 PMC5972899

[22] Deng X, Shi Z, Li S, Wang X, Qiu Y, Shao D, Wei J, et al (2011) Characterization of nonstructural protein 3 of a neurovirulent Japanese encephalitis virus strain isolated from a pig. Virol J 8:209 10.1186/1743-422X-8-209 21549011 PMC3101164

[23] Yamashita T, Unno H, Mori Y, Tani H, Moriishi K, Takamizawa A, et al (2008) Crystal structure of the catalytic domain of Japanese encephalitis virus NS3 helicase/nucleoside triphosphatase at a resolution of 1.8 A. Virology 373:426–436. 10.1016/j.virol.2007.12.018 18201743

[24] Lu G, Gong P (2013) Crystal Structure of the full-length Japanese encephalitis virus NS5 reveals a conserved methyltransferase-polymerase interface. PLoS Pathog 9:e1003549 10.1371/journal.ppat.1003549 23950717 PMC3738499

[25] Ye J, Chen Z, Li Y, Zhao Z, He W, Zohaib A, et al (2017) Japanese encephalitis virus NS5 inhibits type I Interferon (IFN) production by blocking the nuclear translocation of IFN regulatory factor 3 and NF-κB. J Virol 91 pii: e00039-17. 10.1128/JVI.00039-17 28179530 PMC5375679

[26] Pan JR, Yan JY, Zhou JY, Tang XW, He HQ, Xie RH, et al (2016) Sero-molecular epidemiology of Japanese encephalitis in zhejiang, an eastern province of China. PLoS Negl Trop Dis 10:e0004936 10.1371/journal.pntd.0004936 27560360 PMC4999095

[27] Fulmali PV, Sapkal GN, Athawale S, Gore MM, Mishra AC, Bondre VP (2011) Introduction of Japanese encephalitis virus genotype I, India. Emerg Infect Dis 17:319–321. 10.3201/eid1702.100815 21291622 PMC3204761

[28] Tsuchie H, Oda K, Vythilingam I, Thayan R, Vijayamalar B, Sinniah M, et al (1977) Genotypes of Japanese encephalitis virus isolated in three states in Malaysia. Am J Trop Med Hyg 56:153–158. 10.4269/ajtmh.1997.56.153 9080873

[29] Bae W, Kim JH, Kim J, Lee J, Hwang ES (2018) Changes of epidemiological characteristics of Japanese encephalitis viral infection and birds as a potential viral transmitter in Korea. J Korean Med Sci 33:e70 10.3346/jkms.2018.33.e70 29441740 PMC5811662

[30] Sarkar A, Taraphdar D, Mukhopadhyay SK, Chakrabarti S, Chatterjee S (2012) Molecular evidence for the occurrence of Japanese encephalitis virus genotype I and III infection associated with acute encephalitis in patients of West Bengal, India, 2010. Virol J 9:271 10.1186/1743-422X-9-271 23153306 PMC3560186

[31] Schuh AJ, Ward MJ, Brown AJ, Barrett AD (2013) Phylogeography of Japanese encephalitis virus: genotype is associated with climate. PLoS Negl Trop Dis 7:e2411 10.1371/journal.pntd.0002411 24009790 PMC3757071

[32] Huang YS, Hettenbach SM, Park SL, Higgs S, Barrett AD, Hsu WW, et al (2016) Differential infectivities among different Japanese encephalitis virus genotypes in Culex quinquefasciatus mosquitoes. PLoS Negl Trop Dis 10:e0005038 10.1371/journal.pntd.0005038 27706157 PMC5051684

