# Supplementary figures and images for: Differential replication efficiencies between Japanese encephalitis virus genotype I and III in avian cultured cells and young domestic ducklings
Source: PLoS Negl Trop Dis. 2018 Dec 18;12(12):e0007046. doi: 10.1371/journal.pntd.0007046 (PMC6314627; doi:10.1371/journal.pntd.0007046)

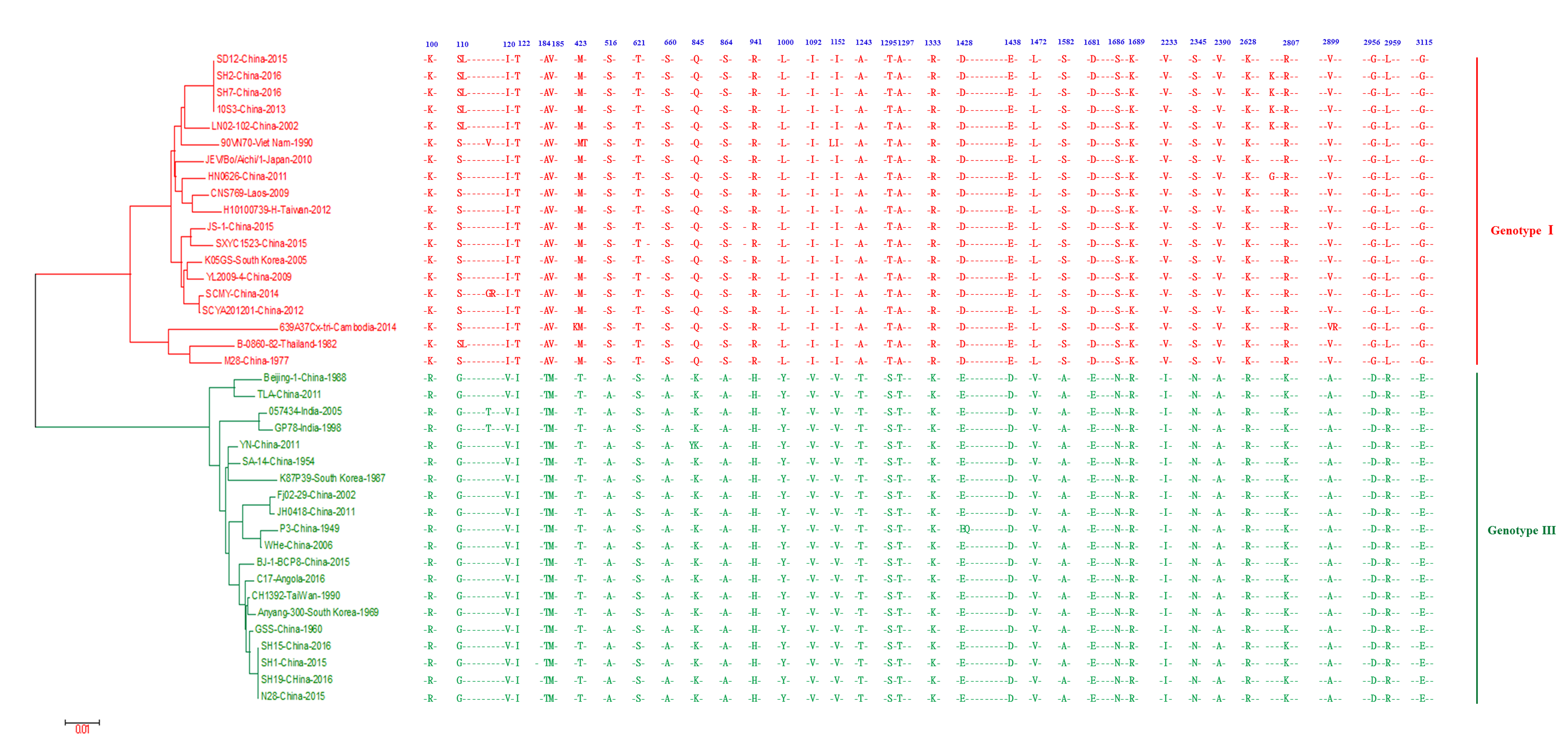

Supplement: S1 Fig — Phylogenetic tree and multiple sequence alignments. The number highlighted in blue indicates the position number of amino acid residue. (TIF) [file pntd.0007046.s001.tif]
