# Supplementary material for: Differential replication efficiencies between Japanese encephalitis virus genotype I and III in avian cultured cells and young domestic ducklings
Source: PLoS Negl Trop Dis. 2018 Dec 18;12(12):e0007046. doi: 10.1371/journal.pntd.0007046 (PMC6314627; doi:10.1371/journal.pntd.0007046)
Supplement: S2 Table — (DOCX) [file pntd.0007046.s003.docx]

**S2 Table 1. Information of JEV strains used for multiple sequence alignment**

| Genotype | Strains | GenBank No. | Source | Date | Country/region |
| --- | --- | --- | --- | --- | --- |
| GI | SD12 | MH753127 | Pig | 2015 | China |
|  | SH2 | MH753133 | *Culex tritaeniorhynchus* | 2016 | China |
|  | SH7 | MH753129 | *Culex tritaeniorhynchus* | 2016 | China |
|  | 10S3 | MF542268.1 | Pig | 2013 | China |
|  | LN02-102 | JF706278.1 | *Culex modestus* | 2002 | China |
|  | 90VN70 | HM228921.1 | *Homo sapiens* | 1990 | Viet Nam |
|  | JEV/Bo/Aichi/1 | AB853904.1 | *Bos taurus* | 2010 | Japan |
|  | HN0626 | JN381837.1 | *Culex* | 2011 | China |
|  | CNS769 | KC196115.1 | *Homo sapiens* | 2009 | Laos |
|  | H10100739/H | KF667324.1 | *Homo sapiens* | 2012 | Taiwan |
|  | JS-1 | KX357114.1 | *Culex tritaeniorhynchus* | 2015 | China |
|  | SXYC1523 | KY078829.1 | *Culex pipiens pallens* | 2015 | China |
|  | K05GS | KR908702.1 | *Culex tritaeniorhynchus* | 2015 | South Korea |
|  | YL2009-4 | JF499789.1 | *mosquito* | 2009 | Taiwan |
|  | SCMY | KU351668.1 | Pig | 2014 | China |
|  | SCYA201201 | KM658163.1 | Pig | 2012 | China |
|  | 639A37Cx-tri | KY927815.1 | *Culex tritaeniorhynchus* | 2014 | Cambodia |
|  | B-0860/82 | GQ902058.1 | Pig | 1982 | Thailand |
|  | M28 | JF706279.1 | *Culex pseudovishnui* | 1977 | China |
| GIII | SH1 | MH753128 | Pig | 2015 | China |
|  | SH15 | MH753130 | *Anopheles sinensis* | 2016 | China |
|  | SH19 | MH753131 | *Anopheles sinensis* | 2016 | China |
|  | N28 | MH753126 | Pig | 2015 | China |
|  | Beijing-1 | L48961.1 | *Homo sapiens* | 1988 | China |
|  | TLA | JN381868.1 | *Homo sapiens* | 2011 | China |
|  | 057434 | EF623988.1 | *Homo sapiens* | 2005 | India |
|  | GP78 | AF075723.1 | *Homo sapiens* | 1998 | India |
|  | YN | JN381871.1 | *Homo sapiens* | 2011 | China |
|  | SA-14 | M55506.1 | Mosquito | 1954 | China |
|  | K87P39 | AY585242.1 | Mosquito | 1987 | South Korea |
|  | Fj02-29 | JF706273.1 | Pig | 2002 | China |
|  | JH0418 | JN381855.1 | *Culex whitmorei and Anopheles sinensis* | 2011 | China |
|  | P3 | U47032.1 | *Homo sapiens* | 1949 | China |
|  | WHe | EF107523.1 | Pig | 2006 | China |
|  | BJ-1-BCP8 | KU871351.1 | unknown | 2015 | China |
|  | C17 | KX945367.1 | *Homo sapiens* | 2016 | Angola |
|  | CH1392 | AF254452.1 | *Culex tritaeniorhynchus* | 1990 | Taiwan |
|  | Anyang-300 | KT447437.1 | Pig | 1969 | South Korea |
|  | GSS | JF706275.1 | Pig | 1960 | China |
