# Supplementary material for: Differential replication efficiencies between Japanese encephalitis virus genotype I and III in avian cultured cells and young domestic ducklings
Source: PLoS Negl Trop Dis. 2018 Dec 18;12(12):e0007046. doi: 10.1371/journal.pntd.0007046 (PMC6314627; doi:10.1371/journal.pntd.0007046)
Supplement: S3 Table — (DOCX) [file pntd.0007046.s004.docx]

**S3 Table. Detection of viremia in JEV-inoculated piglets**

| dpi | GI (SD12)-inoculated piglets | | | | | GIII (N28)-inoculated piglets | | | | |
| --- | --- | --- | --- | --- | --- | --- | --- | --- | --- | --- |
|  | 6980* | 6968 | 1 | 12 | 10 | 6976 | 6071 | 11 | 6067 | 2 |
| 1 | 0 | 0 | 0 | 0 | 0 | 0 | 0 | 0 | 0 | 0 |
| 2 | 2.6±0.2^#^ | 1.1±0.1 | 2.8±0.1 | 1.3±0.1 | 1.5±0.1 | 0 | 0 | 0 | 0 | 0 |
| 3 | 1.4±0.1 | 1.3±0.1 | 1.6±0.1 | 1.1±0.1 | 2.1±0.1 | 2.6±0.1 | 2.8±0.2 | 1.1±0.1 | 1.5±0.1 | 1.7±0.1 |
| 4 | 0 | 0 | 0 | 0 | 3.6±0.1 | 1.4±0.1 | 1.5±0.1 | 0 | 0 | 0 |
| 5 | 0 | 0 | 0 | 0 | 1.6±0.1 | 0 | 0 | 0 | 0 | 0 |
| 6 | 0 | 0 | 0 | 0 | 0 | 0 | 0 | 0 | 0 | 0 |
| 7 | 0 | 0 | 0 | 0 | 0 | 0 | 0 | 0 | 0 | 0 |

*, Ear tag of piglet.

#, Mean ± SD of TCID_50_/ml.

Sixty-day old and antibody-negative piglets (*n*=5) were subcutaneously inoculated with JEV SD12 strain (GI) or N28 strain (GIII) at 5×10^4^TCID_50_ per animal. Blood samples were collected at 1-7 dpi and the viremia levels were measured by TCID_50_ assay.
